# Does anti-HPA-1a affect birthweight in fetal and neonatal alloimmune thrombocytopenia?

**DOI:** 10.1002/pbc.30835

**Published:** 2024-01-11

**Authors:** Margaret McKelvy, Srishti Tyagi, Emilie Vander Haar, Madhavi Lakkaraja, Tim Tomy, Stacy Corke, Thea Palmer, Amihai Rottenstreich, Rick Kapur, Huiying Zhi, Debra Newman, Nina Scatz-Siemers, James Bussel

**Affiliations:** 1 Division of Maternal-Fetal Medicine, Department of Obstetrics and Gynecology, Weill Cornell Medicine, New York, New York, USA; 2 Norton College of Medicine, SUNY Upstate Medical University, Syracuse, New York, USA; 3 Department of Pediatrics, Fred Hutchinson Cancer Center, Department of Pediatrics, University of Washington School of Medicine, Seattle, USA; 4 Department of Pediatrics, Hurley Medical Center, Flint, Michigan, USA; 5 Natibabies.org, Penzance, UK; 6 Department of Obstetrics and Gynecology, Laboratory of Blood and Vascular Biology, Rockefeller University, New York, New York, USA; 7 Division of Maternal- Fetal Medicine, Department of Obstetrics and Gynecology, Zucker School of Medicine at Hofstra/Northwell, New York, New York, USA; 8 Department of Experimental Immunohematology, Sanquin Research, and Landsteiner Laboratory, Amsterdam UMC, University of Amsterdam, Amsterdam, The Netherlands; 9 Department of Pathology, Versiti Blood Center of Wisconsin, Blood Research Institute, Milwaukee, Wisconsin, USA; 10 Department of Pathology and Laboratory Medicine, Weill Cornell Medicine, New York, New York, USA; 11 Department of Pediatrics, Weill Cornell Medicine, New York, New York, USA

**Keywords:** FcRn, FNAIT, HPA incompatibility, placental syncytiotrophoblast, platelet disorders, small for gestational age

## Abstract

**Background:**

Fetal and neonatal alloimmune thrombocytopenia (FNAIT) ensues from parental incompatibility for platelet alloantigens with maternal sensitization. HPA-1a/1b incompatibility is the most common cause of FNAIT in Caucasians. Placental villitis and lower birthweight in FNAIT suggest anti-HPA-1a may have effects beyond inducing thrombocytopenia.

**Objectives:**

Does FNAIT secondary to anti-HPA-1a result in smaller newborns and, the corollary, does antenatal management of FNAIT increase birthweight?

**Study design:**

Birthweights of 270 FNAIT-affected newborns from a randomized clinical trial and a NAITbabies.org survey (135 paired siblings) were compared with those of published controls and treated to untreated FNAIT-affected siblings. Birthweights were converted to percentiles to account for gestational age, sex, and role of birth order in birth weight. Body weights of FNAIT-affected and -unaffected pups in a mouse FNAIT model were analyzed.

**Results:**

Untreated siblings in both the clinical trial and NAITbabies.org cohorts were not small, compared with normal controls. However, treated siblings in both cohorts had significantly higher birthweight percentiles compared with their previous untreated affected sibling. After accounting for gestational age, sex, and birth order, increased birthweight percentile in treated compared with the untreated siblings remained significant in both cohorts. FNAIT-affected neonatal mice had lower bodyweights than FNAIT-unaffected pups.

**Conclusions:**

Untreated FNAIT-affected newborns were not small; however, treatment of FNAIT-affected pregnancies increased newborn birthweights despite corrections to account for other factors that might have influenced the results. High dose IVIG is believed to “block” FcRn and lower maternal anti-HPA-1a levels, and thus increase birthweights by reducing levels of maternal anti-HPA-1a and reducing placental villitis.

## 1| INTRODUCTION

Fetal and neonatal alloimmune thrombocytopenia (FNAIT) results from incompatibility between parental human platelet antigen (HPA) types, most commonly HPA-1bb mothers who make anti-HPA-1a.^1–3^ When sensitization occurs, maternal IgG anti-HPA-1a crosses the placenta, accelerates fetal platelet destruction, and inhibits fetal platelet production, often causing severe fetal and neonatal thrombocytopenia. Intracranial hemorrhage occurs in 10–20% of cases and can be fatal or result in significant disability.^4^ Research on FNAIT has focused almost exclusively on fetal and neonatal thrombocytopenia.

Studies have recently reported decreased birthweight in males born of FNAIT-affected pregnancies.^4,5^ Another study reported similar findings in thrombocytopenic neonates whose mothers had HLA antibodies.^6^ Based on studies demonstrating inflammation in placentas from FNAIT pregnancies,^7–9^ the gender-nonspecific explanation for decreased birthweight is speculated to be placental inflammation (e.g., chronic villitis), presumably due to anti-HPA-1a binding to GPIIIa (*β*_3_) expressed on the surface of syncytiotrophoblasts.^10,11^

This study tests the clinical hypothesis that FNAIT secondary to anti-HPA-1a results in smaller newborns, and the corollary hypothesis that antenatal management of FNAIT in HPA-1a-incompatible pregnancies increases birthweight by reducing the effects of anti-HPA-1a. We analyzed data from two FNAIT cohorts: a published FNAIT clinical trial of antenatal treatment and a survey of FNAIT-affected pregnancies.^12,13^ In addition, we included data from a humanized HPA-1a-specific mouse model of FNAIT.^14^

## 2| MATERIALS AND METHODS

### 2.1| Participants

This paper uses data from three sources, all of which were approved by Institutional Review Boards. The “NAITbabies questionnaire” (Weill Cornell Medicine IRB #22-03024546) was approved on May 25, 2022; the “Clinical Trial” (The New York Presbyterian Hospital IRB #0201-592) was approved in April 2001; the “Mouse Model” (Blood Center of Wisconsin IRB #AUA00005664) was approved on November 15, 2016.***

No treatment or intervention of any kind was administered to the humans in this study. The mothers and newborns involved were either part of a previous FNAIT clinical trial or filled out an anonymous questionnaire describing past FNAIT and its subsequent management.

#### 2.1.1| Role of controls

The birthweights of untreated affected siblings were compared with published normal controls to assess potential effects of FNAIT (anti-HPA-1a) on fetal growth and subsequent newborn size.^15,16^ These controls came from a 2013 meta-analysis that revised the 2003 Fenton Preterm Growth Chart after combining six large population-based surveys of size at preterm birth.^15^ The studies included in the meta-analysis revision were from developed countries and had gestational age correction, data percentiles at 24 weeks of gestational age or lower, data separated by sex, a sample of at least 25,000 babies, and data collected from 1987 to 2012.^15^ The data were combined to produce intrauterine growth curves for each sex, which were joined smoothly with the World Health Organization Growth Standard Curves.^15^

#### 2.1.2| Treated versus untreated siblings

First-affected newborns are, almost always, identified as having FNAIT only after birth. In subsequent FNAIT-affected pregnancies, antenatal treatment is administered to the mother to increase the fetal and neonatal platelet counts. Therefore, in this paper, “untreated” is defined as the mother’s not receiving intravenous IgG (IVIG) and/or steroid treatment during pregnancy, while “treated” refers to subsequent pregnancies in which mothers received such antenatal treatment.

#### 2.1.3| Secondary analysis of the published trial

**FIGURE 1:**
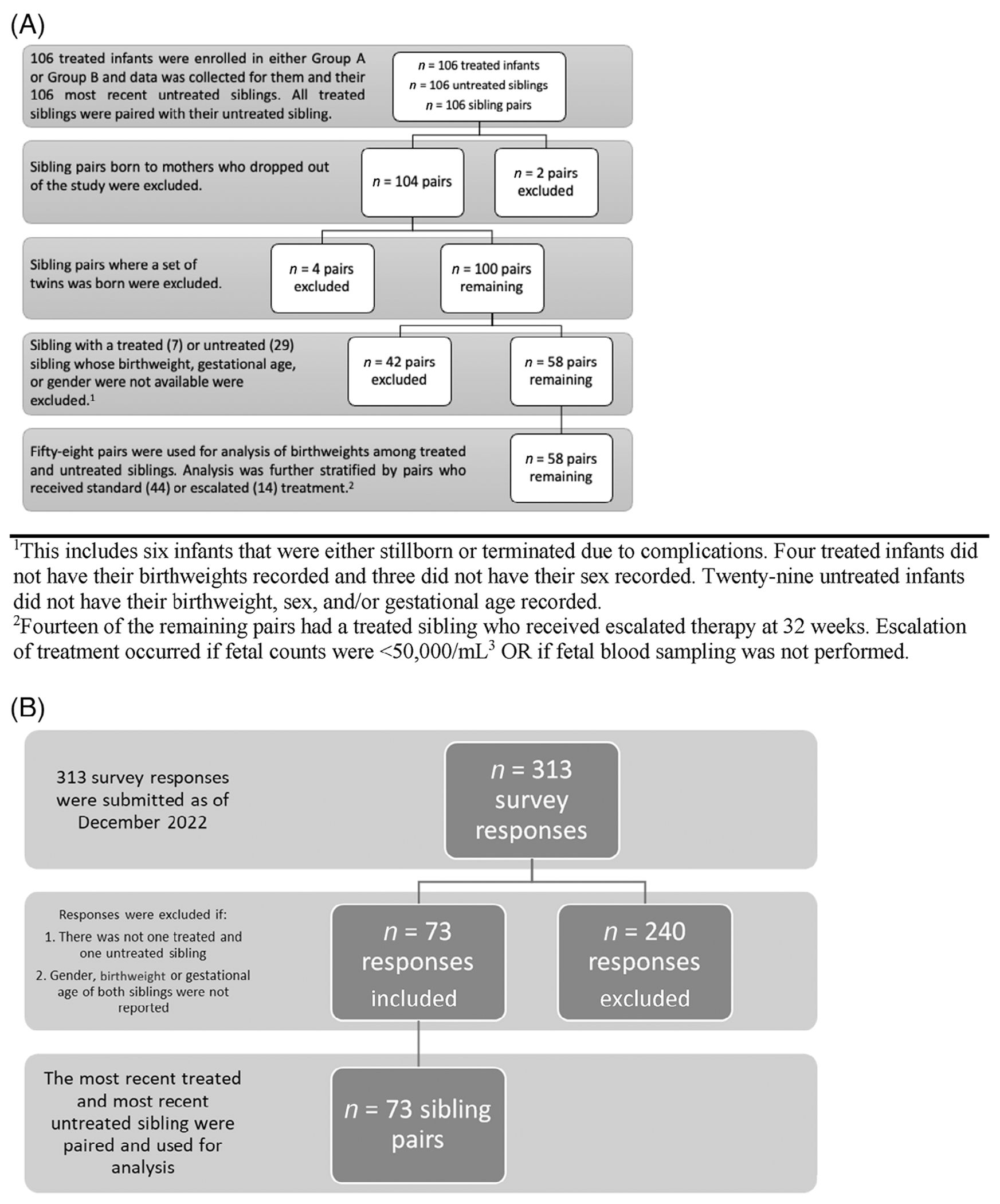
(A) Process of exclusion of sibling pairs from 106 pairs of treated siblings and their most recent, FNAIT-affected and untreated sibling, to the 58 pairs with a treated sibling who received standard (44) or escalated (14) treatment. (B) Process of exclusion of sibling pairs from the 313 NAITbabies questionaries responses to the 73 sibling pairs used for analysis.

From our randomized trial of antenatal management of FNAIT conducted from 2001 to 2013, all pairs of FNAIT-affected siblings, untreated and then treated, were included if their birthweights, gestational ages, and sex had been recorded (Figure 1).^12,13^ Antenatal treatment in the clinical trial was randomized between either IVIG 1 g/kg/infusion twice per week (Group A) or IVIG 1 g/kg/infusion once per week plus prednisone 0.5 mg/kg oral daily (Group B). The clinical trial called for optional fetal blood sampling (FBS) at 32 weeks. Mothers who either had not undergone FBS, or who had but whose fetal platelet count was <50 × 10^6^/*μ*L, per protocol, had their treatment escalated to IVIG twice per week plus prednisone 0.5 mg/kg/day until delivery.

#### 2.1.4| NAITbabies questionnaire

Similar data to that of the clinical trial participants were analyzed from a self-report questionnaire distributed to members of NAITbabies.org in October 2022 regarding pregnancies affected by FNAIT.^17^ All pairs of FNAIT-affected siblings, untreated and then treated, were included if their birthweights, gestational ages, and sex had been reported. Treatment of mothers included IVIG with/without prednisone; exact treatments and doses were not consistently reported and thus not analyzed.

#### 2.1.5| HPA-1a-specific mouse model of FNAIT

To simulate FNAIT-affected and -unaffected neonates in mice, wildtype BALB/c female mice were either left unimmunized or immunized with platelets from HPA-1a-transgenic C57Bl/6 mice to induce HPA-1a-specific antibodies.^14^ Unimmunized and HPA-1a-immunized female mice were bred with either wild-type or HPA-1a-homozygous transgenic C57Bl/6 male mice. Three types of conditions were studied: anti-HPA-1a-negative dams with HPA-1a-expressing pups (control 1), anti-HPA-1a-positive dams with HPA-1a-negative pups (control 2), and anti-HPA-1a-positive dams with HPA-1a-positive pups (experimental). Neonatal platelet counts and birthweights were recorded in all three groups.

### 2.2| Pairing siblings for analysis of treatment effect

The birthweights of untreated and treated siblings from the clinical trial were compared to test the hypothesis that antenatal treatment would lessen the potential effect of anti-HPA-1a, resulting in higher birthweights (Figure 1A).

The NAITbabies data were analyzed similarly. We included all survey responses as of December 2022.

### 2.3| Selection of controls

Birthweights were converted to percentiles incorporating gestational age and biological sex to allow comparison between siblings. Three different birthweight percentile standards from Oken, Aris, and Fenton were evaluated to determine the most suitable one, and the Fenton and Kim publication, representing 3,986,456 births from Germany, United States, Italy, Australia, Scotland, and Canada in a meta-analysis, was selected as the study reference.^15,18,19^ By including data from multiple developed countries, the 2013 Fenton standards increase the generalizability of growth charts compared with previous standards such as the World Health Organization Growth Standard and original 2003 Fenton preterm growth chart.^15^ The PediTools birthweight percentile calculator, a tool based on the 2013 Fenton standards, was used to provide the percentile estimate for specific gestational ages, sex, and birthweights.^16^

### 2.4| Analysis

No a priori power calculations were performed for these secondary *post hoc* analyses. Birthweight percentiles of untreated siblings were compared with published norms.^15,16^ After determining birthweight percentiles, one-sample, two-tailed *t*-tests were conducted to determine whether treated siblings had significantly different birthweight percentiles than their untreated siblings. Comparisons of birthweight percentiles were also made in subgroups based on the biological sex of each treated and untreated sibling, and on the maternal treatment in the clinical trial: IVIG twice per week (Group A) or IVIG once per week with prednisone once per day (Group B). Newborns whose mothers received escalated therapy (IVIG × 2 + prednisone) starting at 32 weeks were included in the overall data set of 58 sibling pairs but also analyzed separately.

Birth order is known to affect birthweight; to compensate for this effect, for first- and second-born sibling pairs (untreated and then treated), we subtracted 130 g from each second-born child delivered at ≥38 weeks and 100 g from each second-born child delivered at <38 weeks.^20,21^ For sibling pairs involving either second- and third-born pairs or third- and fourth-born pairs, we subtracted 50 g from each later-born child delivered at ≥38 weeks and 30 g from each later-born child delivered at <38 weeks.^20,21^ Another approach to elucidate the role of birth order on birthweight percentile involved comparing the birthweights of 42 pairs of siblings in which the untreated and treated pregnancies were of the first- and second-born siblings respectively, with those of 16 sibling pairs in which the affected untreated sibling was a second- (*n* = 13) or third- (*n* = 3) born infant (Table 1); for these latter pairs, the effect of birth order on birthweight is expected to be less (e.g., 50 g instead of 130 g). The same analysis protocol was used to analyze sibling pairs from the NAITbabies questionnaire.

Treatment of subsequent FNAIT-affected pregnancies has been shown to increase newborn platelet counts.^12,13^ A separate analysis explored whether the effects of treatment on neonatal platelet counts paralleled effects on birthweights. To see if there was a correlation between the birth platelet count and the birthweight, differences in birthweight percentiles between treated and untreated newborns were compared with birth platelet counts by simple linear regression analysis (Figure S1). This was also used to compare the difference in birthweight percentiles with the difference in birth platelet counts of the treated and untreated siblings.

Neonatal mouse platelet counts and body weights were analyzed by one-way ANOVA and unpaired two-tailed *t*-tests (Figure 3).

To compensate for performing multiple analyses, a significant difference was defined as *p* ≤ .01, and a trend as *p* ≤ .1.

## 3| RESULTS

### 3.1| Birthweights of FNAIT-affected untreated newborns

#### 3.1.1| Clinical trial

Birthweights of six of 58 (10.3%) untreated neonates were below the 10th percentile (definition of small-for-gestational age): three males and three females (Table S1). The birthweights of 12 (20.7%) males but no females were greater than the 75th percentile; four males were greater than the 97th percentile. Untreated female birthweight percentiles tended to be smaller than untreated male birthweight percentiles (*p* = .07). Overall, untreated FNAIT-affected newborns from the clinical trial, especially males, were not smaller than the normal controls.

#### 3.1.2| NAITbabies questionnaire

Of 73 untreated newborns, the birthweights of nine (12.3%) were below the 10th percentile, including six males and three females (Figure 1B). The birthweights of 15 (20.5%) newborns, 12 males and three females, were above the 75th percentile, including five above the 97th percentile (three female, two male) (Table S1). These untreated FNAIT-affected newborns from the NAITbabies cohort were also not smaller than normal and birthweight percentiles did not differ between males and females.

### 3.2| Birthweights of treated FNAIT-affected newborns

Only three (4.1%) treated newborns, none from the clinical trial, all from the NAITbabies cohort, were <10th percentile for birthweight.

### 3.3| Comparison of untreated and treated FNAIT-affected newborns

#### 3.3.1| Clinical trial

Treated siblings had slightly lower unadjusted birthweights but much lower mean gestational ages than untreated siblings. When birthweights were corrected for gestational age, the 58 treated siblings had higher birthweight percentiles than did their untreated siblings (Table 2). When the 58 pairs of siblings were compared after compensating for birth order by subtracting 50–130 g from the birthweight of the subsequent (treated) sibling (see section Materials and Methods), the treated siblings still tended to be larger (55th percentile) than the untreated siblings (48th percentile) (*p* = .039; Table 1).

#### 3.3.2| NAITbabies questionnaire

When 73 untreated siblings were compared with their treated siblings, a similar large difference in birthweight percentile was seen as with the clinical trial cohort favoring bigger treated siblings (Table 2). When compensating for birth order, the birthweight percentile of the subsequent treated siblings remained significantly greater than that of their untreated siblings (*p* = .002; Table 1).

### 3.4| Effect of Group A and Group B antenatal treatment on corrected birthweight percentile

#### 3.4.1| Clinical trial

**FIGURE 2:**
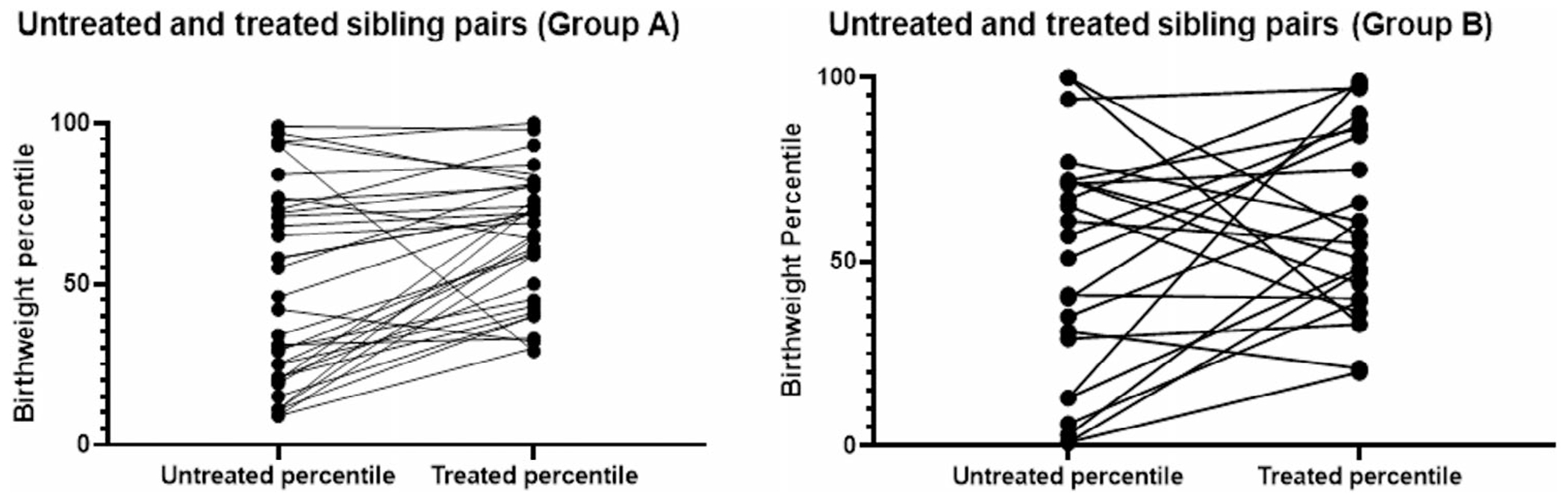
(A) Group A untreated sibling’s birthweight percentile matched to their treated sibling’s birthweight percentile, including those pairs in which the treated sibling received escalated treatment (*n* = 42). (B) Group B untreated sibling’s birthweight percentile matched to their treated sibling’s birthweight percentile, including those pairs in which the treated sibling received escalated treatment (*n* = 29).

There was a trend for treated sibling birthweight percentiles in Group A (IVIG × 2) to be greater than those of the untreated siblings; however, this trend was not seen in Group B (Table 2 and Figure 2). Mothers who received escalated treatment did not have larger treated than untreated infants (*p* = .31; Table 1).

#### 3.4.2| NAITbabies questionnaire

Data on specific treatments, for example, prednisone and IVIG, could not be analyzed due to lack of specific treatment information.

### 3.5| Birthweight percentile and platelet count

There was no correlation between birth platelet count and birthweight percentile in either Group A (*R*^2^ = 0.011) or Group B (*R*^2^ = 0.0054) (Figure S1), and no correlation between birthweight percentile differences and birth platelet count differences in treated and untreated siblings in either treatment group.

### 3.6| Effect of maternal HPA-1a-specific antibodies on birthweights in a mouse model of HPA-1a-specific FNAIT

**FIGURE 3:**
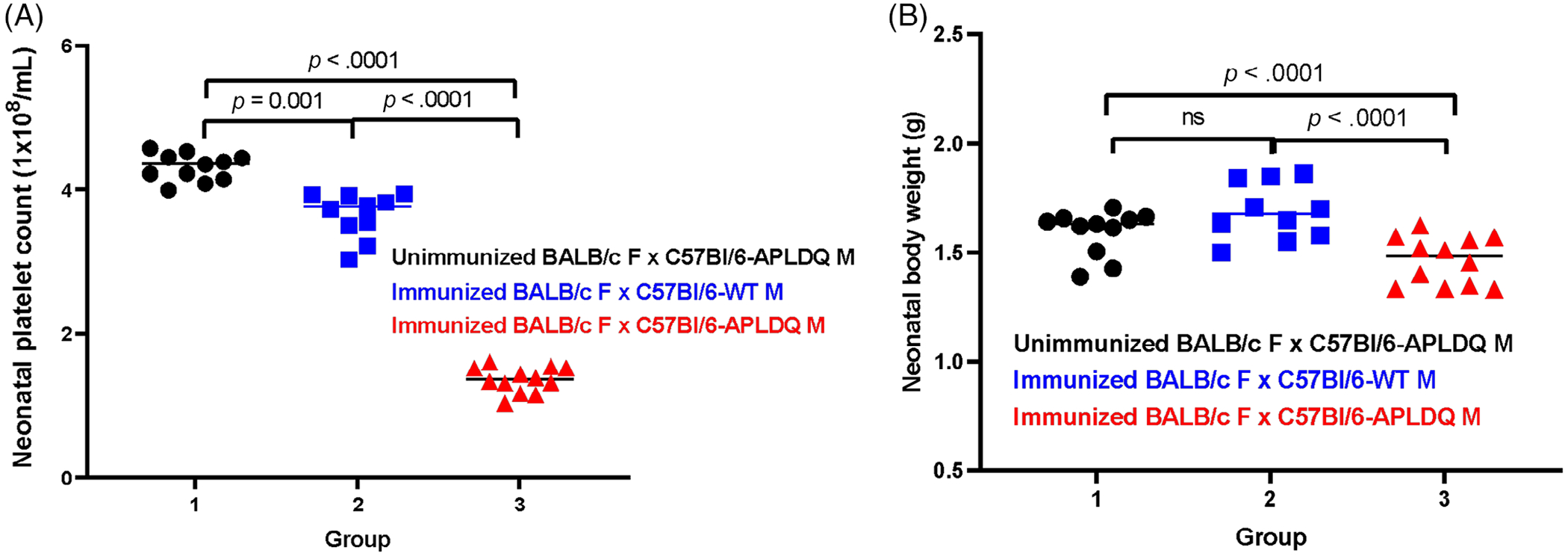
(A) FNAIT affects only pups born toWT BALB/c females that are immunized with C57BI/6-APLDQ platelets and then bred with C57BI/6-APLDQ males. (B) Pups with FNAIT have lower birthweights than do pups without FNAIT.

Neonatal platelet counts were dramatically and significantly lower in HPA-1a-positive pups born to dams with HPA-1a-specific antibodies than in HPA-1a-positive pups born to dams without HPA-1a-specific antibodies or in HPA-1a-negative pups born to dams with HPA-1a-specific antibodies (*p* < .0001; Figure 3A). Importantly, HPA-1a-positive pups born to females with HPA-1a-specific antibodies had substantially and significantly lower neonatal body weights than did HPA-1a-positive pups born to dams without HPA-1a-specific antibodies (*p* = .0092; Figure 3B) or HPA-1a-negative pups born to dams with HPA-1a-specific antibodies (*p* = .0003).

## 4| DISCUSSION

The findings from two large FNAIT cohorts address the question of whether neonates with untreated FNAIT secondary to maternal/fetal HPA-1a-incompatibility may have mildly reduced birthweights. The findings in the 135 untreated newborns from both cohorts neither give evidence that FNAIT-affected babies are small nor that males are smaller than females. However, the treated siblings from both cohorts, another 135, are significantly larger than the untreated ones, even with several adjustments to eliminate confounders. This increased birthweight with treatment raises the possibility that anti-HPA-1a, whose effects would be reduced by maternal treatment, makes babies smaller. The finding of increased size from treated pregnancies occurring primarily in the IVIG × 2 treatment group (Figure 2) implies, based on findings from a placental perfusion study, that the effect is mediated by inhibition of FcRn. This could be by saturation of FcRn with an excess of IgG (functional inhibition) both maternal (lowering levels of anti-HPA-1a in the maternal circulation) and placental/fetal (reducing passage of anti-HPA-1a into the fetal circulation).

**FIGURE 4:**
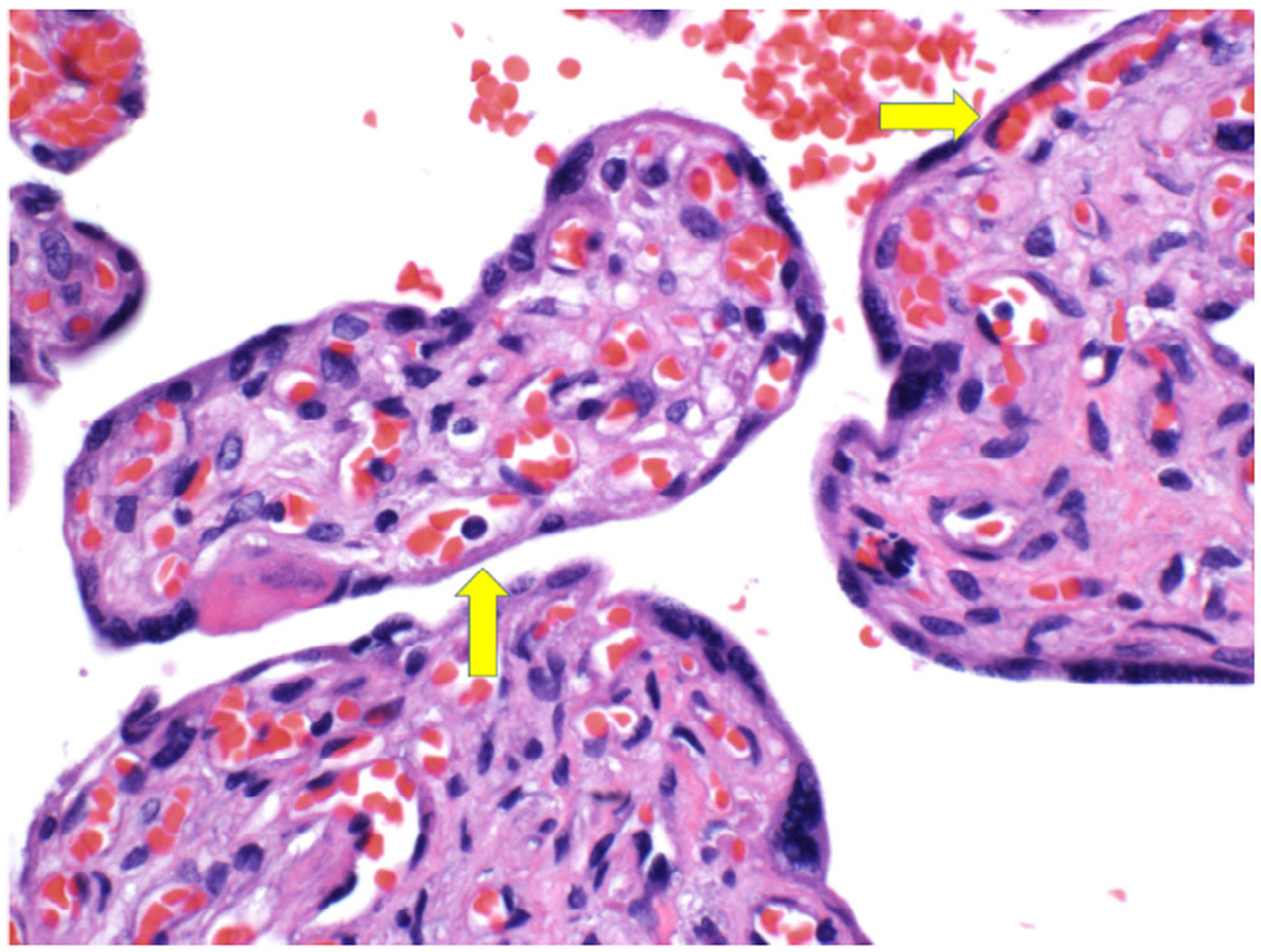
The yellow arrows point to the rim of syncytiotrophoblast between maternal circulation (white space between villi) and fetal circulation (vessels within villi).

Why would anti-HPA-1a reduce birthweight? First, as evidence that this may indeed occur, two studies found that males with anti-HPA-1a-mediated FNAIT are smaller than normal. Second, HPA-1a, expressed on *β*_3_, is on syncytiotrophoblasts in the placenta (Figure 4) and thus is accessible to anti-HPA-1a in the maternal circulation; anti-HPA-1a does not need to enter the fetal circulation to interact with *β*_3_. Third, there are four controlled studies^7–9,22^ demonstrating an increased degree of placental inflammation in anti-HPA-1a-mediated FNAIT; one of these studies demonstrated complement on the placenta implying that IgG antibodies had bound to the placenta. Another small study found that fetuses whose mothers received antenatal treatment had less inflammation.^8^ A recent large study of newborns with FNAIT showed that some babies, especially males again, were small.^23^ A separate study in FNAIT concluded, after exploration of levels of procalcitonin, sFlt1, and CD14, that placental rather than systemic inflammation appeared to modulate the severity of FNAIT.^24^ One way to synthesize this information is that anti-HPA-1a in FNAIT binds to HPA-1a expressed on syncytiotrophoblasts from the fetal part of the placenta causing villitis, which may in turn result in smaller babies. Marked placental inflammation has been found in, for example, preeclampsia to result in smaller babies. Whether the degree of placental inflammation in FNAIT is sufficient to have such an effect is unknown.

Several factors prevented straightforward comparison of previously untreated sibling birthweights with those of subsequently treated sibling birthweights including: elective earlier delivery in the treated siblings, and that second-born siblings are typically larger than first-born siblings. These factors were mitigated by compensations as described in section Materials and Methods and Results.^16,20,21^ Comparing the first- and second-born groups with adjustments, treated siblings nonetheless still tended to be larger than untreated siblings (*p* = .039) based almost solely on the findings in Arm A (IVIG × 2). The NAITbabies data was even stronger for treated siblings being larger (*p* =.002) but specific treatment effects could not be pinpointed. These results create a dilemma: untreated babies were not small but treated babies were significantly larger.

A factor potentially responsible for the increased size of treated neonates would be a direct effect of IVIG and/or prednisone on fetal size through a mechanism unrelated to FNAIT. Review of systemic prednisone use in pregnancy found remarkably little evidence of direct effect on birthweight,^25^ not surprising given that prednisone is almost completely inactivated by 11-beta-hydroxysteroid dehydrogenase in the placenta.^26^ Two studies looking at the effects of antenatal maternal IVIG treatment in other disorders also did not demonstrate an effect on birthweights.^27,28^

As indicated, high-dose IVIG (Group A) tended to result in higher birthweights, whereas IVIG + prednisone (Group B) did not (Table 1). A placental perfusion model had suggested that IVIG could substantially (90%) inhibit transplacental passage of anti-D IgG (presumably the same for anti-HPA-1a), the mechanism believed to be directly responsible for alleviating fetal thrombocytopenia.^29^ These data emphasized that the dose of IVIG would need to be 2 g/kg/wk to achieve the IgG levels required to substantially inhibit transplacental IgG passage, presumably by IgG saturation of placental FcRn. High dose IVIG would also likely lower levels of maternal anti-HPA-1a via saturation of maternal FcRn, mediating its placental effects.^30,31,11^ We believe that passage of anti-HPA-1a to the fetus impacts the fetal platelet counts, whereas the placental (birthweight) effects are driven by anti-HPA-1a in the maternal circulation.

Four studies^7–9,22^ have identified increased frequency of placental chronic inflammation in HPA-1a-incompatible FNAIT-affected pregnancies, indicating a likely effect of anti-HPA-1a on the placenta and providing a potential mechanism for low neonatal birthweights in FNAIT. HPA-1a is present on GPIIIa (*β*_3_), which, in addition to platelets, is found on syncytiotrophoblasts and vascular endothelial cells, where it is expressed in association with *α*_V_ instead of *α*_II_ (Figure 4).^32,33^ One study found that 18 of 21 placentas from FNAIT-affected pregnancies exhibited at least one chronic inflammatory lesion compared with seven out of 42 placentas from age-matched controls (*p* = .001).^7^ Another found that lymphoplasmacytic chronic villitis was not seen in treated pregnancies.^8^ A third study identified C4d deposition on syncytiotrophoblasts in 10 of 14 samples from untreated FNAIT cases, including all small for gestational age (SGA) newborns, compared with two of 21 controls, demonstrating likely activation of the classical pathway of complement by IgG binding to the placenta.^9^ The fourth study found chronic histiocytic intervillositis in 41% of FNAIT pregnancies compared with none in the 21 controls.^22,7,34^ Extensive chronic inflammation (e.g., high-grade chronic villitis) is known to result in fetal growth restriction and SGA newborns.^34,35,22,25,9–11^ Whether and how often the incidence of anti-HPA-1a-associated placental chronic inflammation in FNAIT is severe enough to impact fetal birthweight is unknown and merits further study.

Last, in the HPA-1a-specific mouse model of FNAIT, there was a substantial effect of maternal HPA-1a-specific antibodies on neonatal body weights of HPA-1a-positive but not HPA-1a-negative pups (Figure 3), supporting an effect of maternal anti-HPA-1a on birthweights.^14^

Studies of the mouse placentas from these experiments are not completed yet.

## 5| LIMITATIONS

Despite extensive analyses of data from two cohorts comprising 135 untreated siblings with FNAIT and 135 neonates whose mothers were treated during pregnancy for FNAIT, this study has several limitations. One is lack of a concurrent control group, although assessments of birthweight percentiles were made using published norms derived from 4 million births,^15,16^ both groups of FNAIT-affected newborns had similar findings.^12,13,17^ and comparison of untreated with treated newborns used the same controls. Notably, disagreements exist about which controls are optimal for a given study.^36^ Similarly, while birth order effects are universally agreed upon, how to compensate is based primarily on two studies, albeit with large numbers of neonates.^20,21^ Clinical factors influencing birthweight (e.g., smoking) were not specifically addressed; however, such maternal effects on birthweight are typically consistent across pregnancies and almost all would have resulted in smaller babies.^37–41^ The NAITbabies survey data were based on the memories of respondents. Importantly, placental histology was not assessed, nor were levels of anti-HPA-1a in the maternal circulation or its transplacental passage. Finally, mouse and human placentas are different. Nevertheless, the data derived from 270 newborns with FNAIT allowed us to comprehensively analyze birthweights and the effects of intrapartum FNAIT treatment.

The data from our two newborn cohorts with severe HPA-1a-incompatible FNAIT suggest, if anti-HPA-1a does reduce birthweight, the effect is quite modest. The best evidence in favor of such an effect is the increased birthweight following treatment with IVIG × 2/week. The lack of similar effects following IVIG × 1/week and prednisone support an FcRn-mediated mechanism active only with IVIG twice per week thus achieving IgG levels sufficient to 90% saturate FcRn. The findings discussed above and those in previous publications of both birthweights and placentas^7–9,22^ suggest that maternal anti-HPA-1a may affect the placenta. This has important implications for both long-term platelet count-independent effects of FNAIT and for future prophylaxis in which administered anti-HPA-1a could potentially have an effect.^7–9,22^

The frequency and degree of placental inflammation in FNAIT, its pathophysiology, and whether anti-HPA-1a impacts fetal growth and/or has neurodevelopmental effects are important questions being further explored in ongoing studies.^42^

## Supplementary Material

pbc30835 supplemental figure

pbc30835 supplemental table

Additional supporting information can be found online in the Supporting Information section at the end of this article.

## Figures and Tables

**TABLE 1:** Birthweight percentiles by study group and sex (corrected for birth order^a^).

	Mean (*μ*) treated birthweight percentile	Mean (*μ*) untreated birthweight percentile	*t*	DoF	*p* Value^b^
All treated vs. untreated (clinical trial) (58)	55.19	48.43	2.12	57	.*0386*
F treated/F untreated (all treated; clinical trial) (8)	54.38	43.13	1.78	7	.119
M treated/M untreated (all treated; clinical trial) (20)	57.65	51.2	1.21	19	.241
Treated vs. untreated (standard treatment only; clinical trial) (44)	61.55	45.39	4.78	43	.*000021*
Treated vs. untreated (escalated treatment only; clinical trial) (14)	64.43	58.43	1.054	13	.311
Group A treated vs. untreated (clinical trial) (34)	56.85	48.32	2.32	33	.*0266*
Group B treated vs. untreated (clinical trial) (24)	52.83	48.83	0.715	23	.482
First-born untreated vs. second-born treated (clinical trial) (42)	54.93	48.98	1.50	41	.141
Second/third-born untreated vs. third/fourth-born treated (clinical trial) (16)	55.84	47.38	1.75	15	.0998
Treated vs. untreated (NAITbabies questionnaire) (73)	58.37	48.26	3.17	72	.*0022*

a A total of 130 g was subtracted from second-born siblings born ≥38 weeks; a total of 100 g was subtracted from second-born siblings born <38 weeks; a total of 50 g was subtracted from third- or fourth-born siblings born ≥38 weeks; a total of 30 g was subtracted from third- or fourth-born siblings born <38 weeks.

b Statistical significance was determined using a one-sample, two-tailed *t*-test.

Italicized cells indicate a p value < .05.

**TABLE 2:** Mean demographics by group.

	Clinical trial				NAITbabies questionnaire			
	Untreated		Treated		Untreated		Treated	
Raw birthweight (g)	3169.1		Total: 2959.2 Group A: 2951.2 Group B: 2969.5		3180.8		2770.6	

Birthweight percentile	48.6		Total: 61.2 Group A: 62.3 Group B: 59.5		48.3		64.3	

Gestational age (weeks)	38.3		36.5		38.4		35.4	

Sex	20 F	38 M	25 F	33 M	26 F	47 M	38 F	35 M

## Abbreviations:

FBS: fetal blood sampling
FNAIT: fetal and neonatal alloimmune thrombocytopenia
HPA: human platelet antigen
IVIG: intravenous IgG
SGA: small for gestational age
