# Supplementary figures and images for: Does anti-HPA-1a affect birthweight in fetal and neonatal alloimmune thrombocytopenia?
Source: Pediatr Blood Cancer. Author manuscript; Available in PMC 2026 Apr 14. (PMC13078600; doi:10.1002/pbc.30835)

Figure S1 Treated Birthweight Percentile vs. Birth Platelet Count

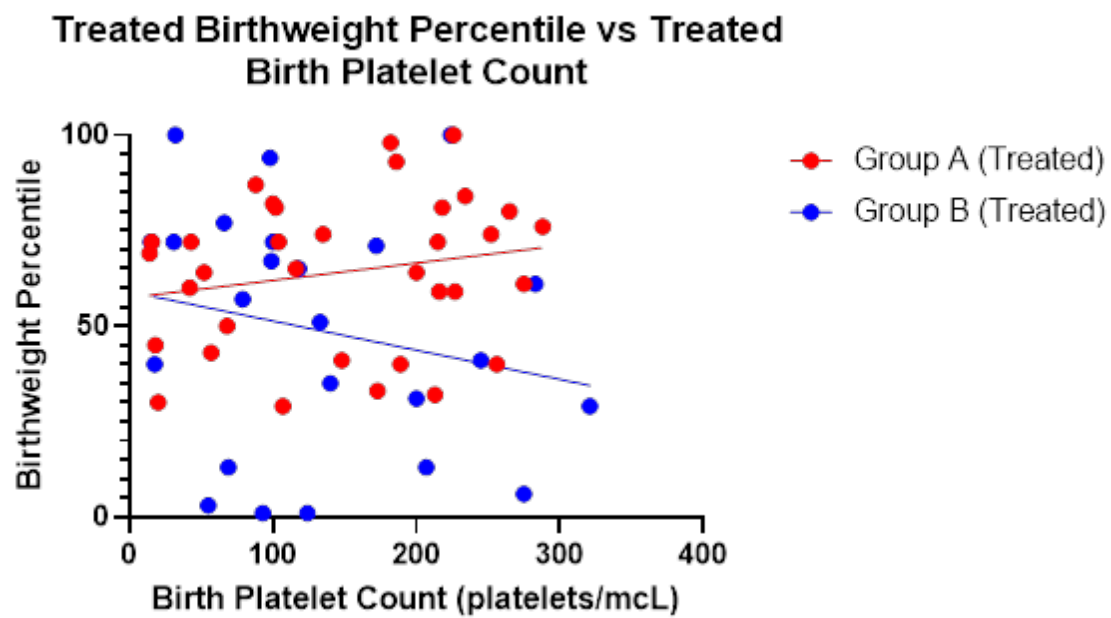

Supplement: pbc30835 supplemental figure [file NIHMS2158741-supplement-pbc30835_supplemental_figure.pdf]
