# Supplementary material for: Does anti-HPA-1a affect birthweight in fetal and neonatal alloimmune thrombocytopenia?
Source: Pediatr Blood Cancer. Author manuscript; Available in PMC 2026 Apr 14. (PMC13078600; doi:10.1002/pbc.30835)
Supplement: pbc30835 supplemental table [file NIHMS2158741-supplement-pbc30835_supplemental_table.docx]

**Supplemental** **Tables and Figures:**

TABLE S1. Contingency Table of Birthweight Percentiles by Cohort and Sex

|  | **Clinical Trial** | | | **NAIT Babies Questionnaire** | | |
| --- | --- | --- | --- | --- | --- | --- |
|  | **Untreated Infants** | **Untreated male*** | **Untreated female*** | **Untreated Infants** | **Untreated male** | **Untreated female** |
| **3rd and below** | 3 | 1 | 2 | 7 | 3 | 4 |
| **4th-10th** | 3 | 2 | 1 | 2 | 2 | 0 |
| **11th-25th** | 12 | 8 | 4 | 10 | 4 | 6 |
| **26th-50th** | 12 | 5 | 7 | 21 | 14 | 7 |
| **51th-75th** | 16 | 9 | 7 | 18 | 12 | 6 |
| **76th-89th** | 4 | 4 | 0 | 8 | 8 | 0 |
| **90th and above** | 8 | 8 | 0 | 7 | 4 | 3 |
| **Total** | **58** | 37 | 21 | **73** | 47 | 26 |
| *p < 0.1 |  |  |  |  |  |  |
